# 

**Published:** 1856-01

**Authors:** 


PUBLISHED MONTHLY, AT $3 PER ANNUM IN ADVANCE.

### ATLANTA
### MEDICAL AND SURGICAL
### JOURNAL

EDITED BY
JOSEPH P. LOGAN, M. D.,
PROFESSOR OF PHYSIOLOGY AND GENERAL PATHOLOGY,
AND
W. F. WESTMORELAND, M. D.,
PROFESSOR OF THE PRINCIPLES AND PRACTICE OF SURGERY.

Pax et scientia, sed veritas sine timore.

VOL. I. JANUARY, 1856. NO. V.

ATLANTA, GEORGIA:
PRINTED BY C. R. HANLEITER AND CO.
1856.

☞ Each Number contains sixty-four pages. POSTAGE.—Under 500 miles, 15 cents a year; over 500 miles, 30 cents a year—to be pre-paid quarterly. If not pre-paid, double the above rates.

